# Concurrent enhancement of provitamin A and yield in tropical maize hybrids

**DOI:** 10.3389/fpls.2025.1611495

**Published:** 2025-07-17

**Authors:** Abebe Menkir, Ibnou Dieng, Silvestro Meseka, Bussie Maziya-Dixon, Bunmi Bossey, Oyekunle Muhyideen, Manfred Ewool, Mmadou Coulibaly, Wende Mengesha

**Affiliations:** ^1^ Research for Development, International Institute of Tropical Agriculture, Ibadan, Nigeria; ^2^ Institute for Agricultural Research, Ahmadu Bello University, Zaria, Nigeria; ^3^ Maize Unit, Crop Research Institute, Kumasi, Ghana; ^4^ Maize Unit, Institute de Economic Rurale, Bamako, Mali

**Keywords:** genetic gain, carotenoids, provitamin A, maize, tropical hybrid

## Abstract

Maize is a strategic food crop in sub-Saharan Africa, where vitamin A deficiency affects millions. Significant investments have thus focused on developing maize varieties that provide 50% of the daily vitamin A requirement for vulnerable populations. Despite the release of many provitamin A enriched maize varieties across Africa, estimates of genetic gains in provitamin A content and grain yield from long-term breeding programs are undocumented. This study analyzed data from 124 provitamin A-enriched hybrids and eight commercial checks recorded over 12 years across diverse environments to estimate genetic gains. Results showed a significant annual increase of 2.05% in provitamin A and 3.54% in β-carotene, alongside a 1.09% reduction in β-cryptoxanthin. Additionally, our breeding program achieved an annual genetic gain of 1.88% in grain yield while simultaneously decreasing silking days by 0.09% and plant aspect score by 0.35%, alongside a 0.31% increase in plant height. We identified 23 hybrids that accumulated 54% to 95% more provitamin A and produced 10% to 30% higher grain yields compared to the best commercial hybrid (COMYH129). These findings demonstrate concurrent genetic gains in both provitamin A content and grain yield, highlighting the potential for further productivity increase and accumulation of beneficial carotenoid to improve human health.

## Introduction

Maize emerged as a prominent food crop and spread rapidly across regions in Africa by the early 1700s following its introduction into the continent in the 16th century (Chemiwchan and Morano-Cruz, 2019). Over the span of 100 years, maize transitioned from being a garden crop to a major staple food, supplying calories, proteins, and other nutrients to millions of people across Africa (McCann, 2005; Smith et al., 2022). This spectacular spread of maize was driven by its competitiveness with indigenous African food crops in terms of calories and nutrients, its high yield potential, the simplicity of its cultivation, low labor requirements, short maturity cycle, ease of storage and transportation, protective husks that shielded the cobs from birds and rain, and the ability to harvest it at different times in a single growing season to meet seasonal food deficits (Purseglove, 1972; McCann, 2005).

Before the 1960s, the introduced maize had varying kernel colors, with many local consumers in Africa favoring foods prepared from colored maize grain, including yellow (McCann, 2005). However, the demand for a cheap source of food to feed mine workers, as well as the increasing need to export maize for breweries and starch industries in Great Britain promoted a significant shift towards the cultivation of white maize in southern Africa, which later spread to other regions of the continent (McCann, 2005). The resulting over-reliance of Africans on white maize-based diets, which provided adequate calories but lacked other nutrients in sufficient quantities, including vitamin A, has adversely affected the overall growth and cognitive development of children and young people throughout their lives (Bailey et al., 2015).

The preference for yellow and orange maize in Africa is anticipated to rise, considering the shift towards white maize that occurred in the 1920s and 1930s (McCann, 2005). Yellow kernel color has been recognized as an important trait in at least one major maize-growing region across all countries in West and Central African, apart from Benin Republic (CIMMYT, 1988). The potential for increased yellow maize cultivation across Africa is strong due to its ability to supply various carotenoids that enhance nutritional benefits, the rising demand for healthy diets and food processing, its appealing flavor and taste (Elemo, 1999; Omueti and Agbaji, 1999; McCann, 2005; Trono, 2019), and its contribution to improving the color of egg yolks, poultry meat, and animal fat (Kaul et al., 2019; Nabi et al., 2020).

Research in white maize-consuming regions of Central and South Africa has revealed an acceptability rate of 45% to 47% for yellow or orange maize, indicating a strong potential for increased consumer adoption (Stevens and Winter, 2008). Additionally, Manjeru et al. (2019) propose that negative consumer perceptions of yellow maize may not apply to provitamin A-enriched orange maize, even in areas where white maize is the primary food source. In this context, promoting the production and consumption of provitamin A-enriched orange maize among low-income households lacking access to diverse diets (Msungu et al., 2022) could aid in disease prevention and mitigate the adverse effects of vitamin A deficiency. As a result, significant investments have been made for nearly two decades to develop tropical and sub-tropical orange maize varieties with up to tenfold increase in provitamin A content and acceptable yields (Pixley et al., 2013; Bouis and Saltzman, 2017; Menkir et al., 2017; Prasanna et al., 2020).

Baudron et al. (2024) demonstrate that adopting provitamin A-enriched maize hybrids could meet 50% of the daily vitamin A requirements for many households in production areas where provitamin A levels are low. Furthermore, the provitamin A in these crop varieties is bioavailable and can improve the vitamin A status of vulnerable populations (Dwivedi et al., 2023). As a result, millions of people who rely on maize, particularly women and children, could benefit from consuming orange maize enriched with provitamin A carotenoids. The intake of carotenoid-rich foods is linked to a reduction in debilitating diseases and an increase in various health benefits (Menkir et al., 2008; Yuan et al., 2023).

In many maize-growing regions of sub-Saharan Africa, where farming communities face vitamin A deficiencies and low incomes resulting from low agricultural productivity, the development and deployment of maize varieties that combine high provitamin A content with high yield potential can enhance income and optimize nutritional benefits for rural and urban households. Considerable efforts have thus been made over the decades, leading to the successful development and release of provitamin A-enriched maize varieties that yield competitively with commonly grown cultivars. Despite these achievements, the perception of a trade-off between enhancing vitamin A content and crop productivity persists in the literature as a potential barrier that limits farmer adoption of nutritious crop varieties (van Ginkel and Cherfas, 2023). While some studies have found a link between low grain yields and improvements in provitamin A content in maize (Senete et al., 2011; Dhliwayo et al., 2014; Ortiz et al., 2019), other studies have reported that increasing provitamin A had either positive (Halilu et al., 2016; Khamkoh et al., 2019; Prasanna et al., 2020; Menkir et al., 2021) or neutral (Menkir et al., 2014; Suwarno et al., 2014; Zanga et al., 2016; Duo et al., 2021) effects on grain yield. These contradictory findings may arise from data generated in trials involving a limited number of genotypes developed over a short breeding period and tested across few locations and years. Consequently, use of data collected from trials that evaluate many genotypes across diverse locations for an extended period may provide stronger evidence for the feasibility of achieving simultaneous genetic improvements in provitamin A content and grain yield.

Genetic gain studies have reported reductions in nitrogen (N), phosphorus (P), potassium (K), copper (Cu), and manganese (Mn) concentrations in the grain of wheat cultivars as grain yield increases (Calderini et al., 1995; Ortiz-Monasterio et al., 1997; Ekholm et al., 2007; Murphy et al., 2008; Hao et al., 2022). Similarly, decreasing concentrations of iron (Fe) and zinc (Zn) in wheat grain were found with rising yields (Fan et al., 2008; Murphy et al., 2008; Amiri et al., 2018). In contrast, significant positive improvements in both grain yield and Fe and Zn concentrations have been observed when breeding efforts prioritize simultaneous improvements in yield potential and nutrient content (Govindan et al., 2022). Nevertheless, estimates of genetic gains for both grain yield and provitamin A content obtained from analyses of long-term trials have not been documented. Furthermore, the impact of a long-term breeding approach on changes in the concentrations of other beneficial carotenoids and desirable agronomic traits has not been assessed, which is crucial for guiding future improvements in productivity and human nutrition.

Given the high costs associated with breeding for both enhanced concentrations of beneficial carotenoids and superior agronomic performance to produce appealing products, periodic evaluation of the rate of realized genetic gains for these traits is necessary to assess the effectiveness of the current breeding strategy to achieve the desired improvements. In most recent studies, historical trial data containing many genotypes including benchmark varieties has been used to generate reliable genetic gain estimates for grain yield and other traits (Diang et al., 2024). Such studies are crucial to guide the establishment of new breeding approaches for achieving significantly higher genetic gains in productivity and nutritional quality to meet the increasing demands for maize grain in the next 20–30 years (Virk et al., 2021; Smith et al., 2022: Diang et al., 2024). The present study was therefore conducted to (i) estimate realized genetic gains achieved through selection for high provitamin A content and desirable agronomic traits in tropical maize hybrids, (ii) understand the associated changes in beneficial carotenoids and yield-related traits, and (iii) assess the potential in identifying hybrids with elevated levels of provitamin A and high grain yields for commercialization.

## Materials and methods

### Hybrids evaluated in collaborative regional trials

The present study utilized historical data sets of 132 hybrids evaluated in collaborative regional trials across diverse locations from 2010 to 2021 to estimate the realized genetic gains. These trials involved 124 elite provitamin A-enriched (EPVA) hybrids along with four yellow/orange commercial hybrids (COMY), three released provitamin A enriched (RPVA) hybrids, and a farmer-preferred yellow (FPYC) cultivar as benchmark checks (**Supplementary Table S6**). The parental lines of the elite hybrids were developed through repeated inbreeding with selection for desirable agronomic and adaptive traits, and kernel color and texture at each inbreeding stage (Menkir et al., 2017). At the S5 inbreeding stage, the most promising lines were screened for provitamin A and other carotenoids using high-performance liquid chromatography (HPLC). The breeding strategy used to develop the lines with intermediate to high levels of provitamin A that formed the elite hybrids was described in detail by (Menkir et al., 2017). The COMYH and RPVA hybrids marketed in Nigeria were obtained from national, regional, and multinational seed companies. The FPYC represented a recycled hybrid maize commonly grown around the testing site where the collaborative regional trials were conducted.

Every year, IITA has shared collaborative regional trials involving elite provitamin A-enriched hybrids along with commercial checks for regional testing in partnership with national partners and private seed companies across many locations. These collaborative regional trials were also evaluated at IITA experiment stations representing the diverse agro-ecological zones in West and Central Africa to assess agronomic performance of hybrids and collect samples for carotenoid analyses. The data recorded in collaborative regional trials conducted across locations from 2010 to 2021 were thus considered suitable for estimating genetic gains in the present study (Eberhart, 1964; Diang et al., 2024). The dynamics of continually adding new hybrids and removal of inferior ones from the trials each year resulted in a total of 16 to 48 hybrids being evaluated. Annually, 2 to 21 new hybrids were then consistently added, while hybrids exhibiting poor agronomic performance in the previous year were removed. In the collaborative regional trials, four EPVA, one COMYH, and two RPVA hybrids were tested for nine years to maintain the connectivity in the data sets used in the present study. Additionally, 67 EPVA and three benchmark hybrid checks were advanced and re-evaluated for two to eight years, making the data sets well connected to the preceding testing years. The good connectivity of these data sets could thus allow a robust assessment of genetic gain estimates in our study.

### Performance testing in multiple environments

A total of 16 to 48 hybrids included in the collaborative regional trials were arranged in appropriate alpha lattice designs and planted with three replications during the main rainy seasons in collaboration with partners in the national agricultural research systems (NARS) and private seed companies (SMEs) in 16 to 41 testing locations in Benin Republic, Cameroon, Democratic Republic of the Congo (DRC), Egypt, Ethiopia, Ghana, Mali, Nigeria, and Uganda during the 12-year period. These test locations captured different soil types, weather conditions, length of the growing period, a broad range of management practices, and planting dates. The plot size used for each hybrid was one row of 5 m length spaced 0.75 m apart with a spacing of 0.5 m between plants within a row. At the International Institute of Tropical Agriculture (IITA) testing sites in Nigeria, two seeds were placed in each hill and later thinned to one plant after emergence to attain a population density of 53,000 plants per hectare. A compound fertilizer was applied at the rate of 60 kg N, 60 kg P, and 60 kg K per hectare at planting, followed by an additional application of urea at the rate of 60 kg N per hectare four weeks later. The trial fields in these testing sites were sprayed with gramazone and atrazine as pre-emergence herbicides at the rate of 5 L per hectare, followed by manual weeding to keep the trials weed-free. The collaborators in the NARS and SMEs used recommended crop management practices, rates of fertilizer applications, and weed control methods for each of their regional trial testing locations.

### Sample collection and carotenoid analyses

The collaborative regional trials were conducted at six locations in Nigeria (Ibadan, Ikenne, Kadawa, Mokwa, Saminaka, and Zaria) and three locations in Ghana (Fumesua, Kpeve, and Kwadaso) over a period of 12 years. The number of testing locations varied from four to seven during the evaluation of these trials during the 12-year period. For each hybrid included in the trials, four representative plants were self-pollinated in the first two replications to protect the ears from contamination with pollen blown from other maize hybrids. The covered ears harvested at each location were carefully threshed to form composite samples for carotenoid analysis at the Crop Utilization Laboratory of the International Institute of Tropical Agriculture using High-Performance Liquid Chromatography (HPLC). The protocol described by Howe and Tanumihardjo (2006) was used to extract the carotenes (α- and β-carotenes) and xanthophylls (β-cryptoxanthin, lutein and zeaxanthin) from the grain samples and perform the analyses with HPLC. Provitamin A was calculated by summing the concentrations of β-carotene (including all-trans, 13-cis, and 9-cis isomers), and half concentrations of each of β-cryptoxanthin and α-carotene, since β-cryptoxanthin and α-carotene can provide only one molecule of retinol each as opposed to two molecules of retinol for β-carotene (Institute of Medicine, 2012).

### Agronomic trait measurements

In the present study, seven important agronomic traits associated with desirable hybrid performance were measured. The number of days from planting to when 50% of the plants in each plot were shedding pollen and had emerged silks were recorded as anthesis days and silking days, respectively. We measured plant height as the distance from the base of the plant to the height of the first tassel branch, and ear height as the distance from the base of the plant to the node bearing the upper ear in centimeters. Husk cover was rated on a scale of 1 to 5, where 1 indicates husks tightly arranged and extended beyond the ear tip, and 5 indicates exposed ear tips. Plant aspect was visually rated on a scale of 1 to 5, where 1 represents a desirable plant type with large and similar ears, low ear placement, shorter plants, resistance to tropical foliar diseases, and little stalk and root lodging, and 5 represents plants with small and variable ears, high ear placement, tall plants, susceptibility to tropical foliar diseases, and stalk and root lodging. We visually rated ear aspect on a 1 to 5 scale, where 1 indicates clean, uniform, and large ears, and 5 indicates rotten, variable, and small ears. After harvest, representative ears from each plot were shelled to determine the grain moisture content. Grain yield was then calculated from the ear weight adjusted to 15% grain moisture, assuming a shelling percentage of 80% in each plot.

### Realized genetic gain estimates

The realized genetic gain analysis was conducted using 62 year-location combinations (environments) for carotenoids and 307 year-location combinations (environments) for agronomic traits. The addition of new hybrids and the removal of inferior ones in the collaborative regional trials resulted in unbalanced data sets for estimating genetic gains in carotenoids and agronomic traits. We used a three‐step mixed‐model approach that explicitly accommodates unbalanced data following Diang et al. (2024). In the first step, each trial evaluated in an environment was analyzed with a spatial mixed model to adjust for field trends, in which genotype, block, and replication were considered as independent random effects, to estimate broad‐sense heritability (repeatability) values (Smith et al., 2001; Cullis et al., 2006). Estimating genetic gain involves identifying a genetic and a nongenetic trend in a Linear Mixed Model. When hybrid and year are considered as independent random main effects, it generates biased outcomes because shrinkage will lead to underestimation of genetic effects. Treating genotypes and years as fixed effects was thus necessary to account for potential nonlinear trends in both genotype and year effects (Mackay et al., 2011; Diang et al., 2024). Environments with repeatability values of less than 0.20 were 5 for lutein, 4 for zeaxanthin, 5 for β-cryptoxanthin, 12 for α-carotene, 6 for β-carotene, and 4 for provitamin A. Additionally, there were 66 environments for anthesis days, 67 for silking days, 72 for plant height, 85 for ear height, 135 for husk cover, 72 for ear aspect, 103 for plant aspect, and 53 for grain yield with repeatability values below 0.20. Environments with low repeatability values indicate less reliable hybrid performance in trials and may not accurately reflect the true genetic potential of the hybrids being evaluated for their genetic effects. Consequently, these environments were excluded from the analyses of the two data sets to improve our understanding of hybrid genetic potential under more reliable and consistent conditions for obtaining more accurate genetic gains estimates.

In the second step, best linear unbiased estimates (BLUEs) were computed for hybrids evaluated in each environment along with their prediction error variances. The resulting BLUEs of the hybrids for the selected environments were then subjected to combined weighted linear mixed model analyses, using weights equal to the inverse of each BLUE’s prediction‐error variance (Cullis et al., 2006; Diang et al., 2024). In the final step, realized genetic gains were calculated using a weighted linear regression of the combined BLUEs of the hybrids on the year of hybrid origin, which was defined as the first year a hybrid was included in the collaborative regional trial. The percentage change in genetic gain for each carotenoid including provitamin A, and agronomic traits attributed to genetic causes was estimated as a ratio of the regression slope to the y-intercept of the regression plus the slope multiplied by the year of first testing (Cullis et al., 2006). Statistical significance of the slope was assessed using the Student’s t-test which evaluates whether the estimated slope differs from zero based on two-sided p-value.

### Genetic correlations

Genetic correlations were computed for each pair of carotenoids or agronomic traits following a two-stage method of analysis proposed by Smith et al. (2001). In the first stage, we conducted single year-location analysis to estimate the BLUEs within each environment and their corresponding weights for each carotenoid and agronomic trait, which are the inverse of the squared standard errors of the hybrid BLUEs. In the second stage, we performed a multi-trait linear mixed model analysis for the combined model. We modeled the Genotype-by-Environment (GxE) as nested effects considering a general heterogeneous correlation structure.

### Evaluation of changes in carotenoid and agronomic trait profiles in hybrids

To examine changes in carotenoid profiles and agronomic traits occurring in hybrids, principal component analyses was computed using the correlation matrix of (i) hybrid BLUEs for carotenoids (lutein, zeaxanthin, β-cryptoxanthin, α-carotene, β-carotene) and (ii) agronomic traits (anthesis, silking, plant height, ear height, husk cover, plant aspect, ear aspect) to stratify the hybrids into groups based on Ward’s clustering method (Ward, 1963). The resulting cluster groups, along with the principal component axis scores for carotenoids or agronomic traits, were used to perform canonical discriminant analyses to visualize differences among groups using the CANDISC procedure in SAS (SAS Institute, 2021). Simple correlation analysis was conducted between hybrid BLUEs for carotenoids and agronomic traits and the corresponding scores for the three canonical discriminant functions (CAN1, CAN2, and CAN3) to identify the carotenoids or agronomic traits that significantly contribute to each function. Group means averaged across hybrid BLUEs were calculated using the univariate procedure in SAS (SAS Institute, 2021). To further assess the concurrent changes in both carotenoids and agronomic traits, correlation analyses were performed: (i) between the hybrid BLUEs of each carotenoid and each of the three CAN scores for agronomic traits, and (ii) between the hybrid BLUEs of each agronomic trait and each of the three CAN scores for carotenoids, all using SAS (SAS Institute, 2021).

## Results

### Genetic gain estimates for provitamin A and other carotenoids

As lutein and zeaxanthin account for over 75% of the total carotenoids in yellow and orange tropical maize inbred lines and common cultivars (Yuan et al., 2023), our breeding approach has focused on enhancing the concentrations of provitamin A carotenoids in the grains. The provitamin A-enriched hybrids developed over the years were then evaluated in regional trials across 62 test environments, with average provitamin A content ranging from 1.5 to 12.4 µg/g (**Supplementary Table S1**). Nearly 60% of these environments had average provitamin A content exceeding 8.0 µg/g. The repeatability values for provitamin A content recorded in 82% of the test environments ranged from 0.52 to 0.98, indicating that the hybrid effect was strong (**Supplementary Table S2**). Mean provitamin A content varied from 1.5 to 12.4 µg/g for EPVA hybrids, 2.5 to 5.9 µg/g for COMY hybrids, and 8.9 to 10.1 µg/g for RPVA hybrids (**Supplementary Table S2**). As a group, the EPVA hybrids accumulated 97% more provitamin A than the COMY hybrids in collaborative regional trials. More than 80% of the EPVA and three RPVA hybrids accumulated 30 to 47% provitamin A carotenoids in their kernels, whereas the COMY hybrids and FPYC cultivar accumulated only 11 to 29% provitamin A carotenoids (**Supplementary Table S1**). Interestingly, lutein and zeaxanthin constituted at least 60% of the total carotenoids in most EPVA and RPVA hybrids. Realized genetic gain estimates revealed a significant (*P*<0.0001) linear annual increase of 0.17 µg/g in provitamin A, translating to a relative annual genetic gain of 2.05% (**Table 1**). Additionally, there was a significant (*P*<0.0001) yearly increase of 3.54% in β-carotene, while β-cryptoxanthin experienced a significant (*P*<0.05) reduction of 1.09% per year.

**Table 1:** Genetic gain estimates for provitamin A content and different carotenoids of hybrids evaluated in collaborative regional trials for 12 years.

Carotenoids	(%)	Slope
Lutein (µg/g)	0.17	0.014
Zeaxanthin (µg/g)	-0.09	-0.014
β-cryptoxanthin (µg/g)	-1.09	-0.054*
α-carotene (µg/g)	-0.18	-0.002
β-carotene (µg/g)	3.54	0.182****
Provitamin A (µg/g)	2.05	0.166****

Slops marked with * and **** are significantly different at *P*<0.05 and *P*<0.0001 levels, respectively.

### Effects of improvements in provitamin A on different carotenoids

**Figure 1:**
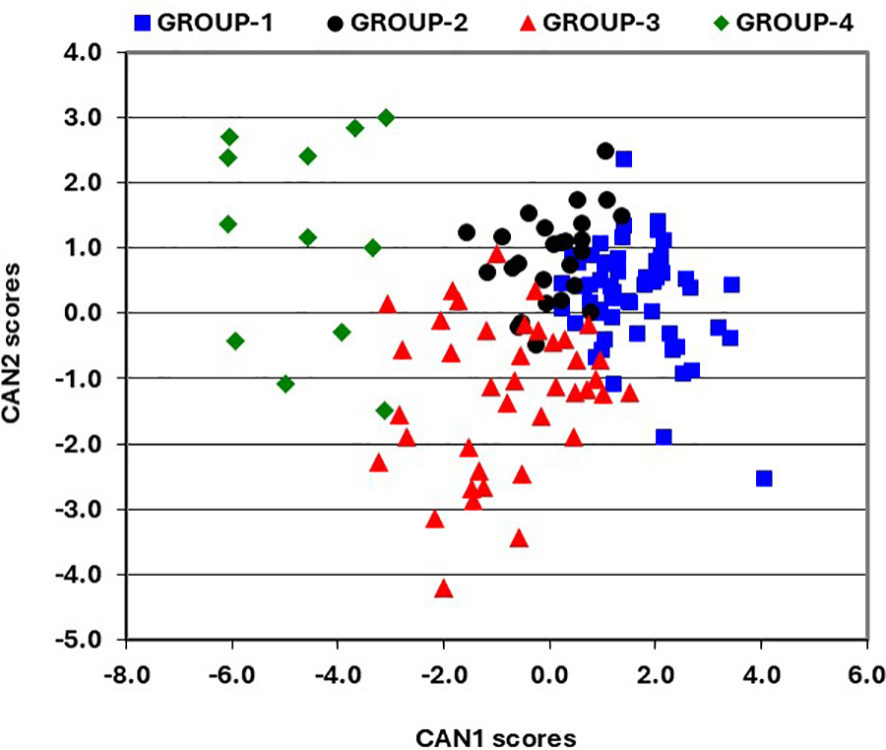
Groups of lines classified using Ward’s clustering method, aligned with a scatter plot of the first and second canonical discriminant function scores for carotenoids.

The impact of improvements in provitamin A accumulation on the concentrations of all carotenoids in hybrids was assessed using genetic correlations (**Supplementary Table S3**). The genetic correlations for pairs of carotenoids produced in both the α- (lutein and α-carotene) and β- (β-carotene, β-cryptoxanthin, and zeaxanthin) branches of the carotenoid biosynthetic pathway were significant and positive, although they were not strong. Notably, α-carotene exhibited a positive correlation with β-carotene, β-cryptoxanthin, and zeaxanthin. In contrast, lutein displayed the weakest genetic correlations with other carotenoids. Provitamin A was positively correlated with all carotenoids except lutein. Further assessment of carotenoid profiles and content in hybrids was made using canonical discriminant analysis of principal component axes scores. The resulting three discriminant functions (CAN1, CAN2, and CAN3) explained 72%, 17%, and 12% of the variation in carotenoids among hybrids, respectively (**Table 2**). CAN1 exhibited genetic changes that significantly (*P*<0.0001) increased provitamin A accumulation, mainly through marked rises (*P*<0.0001) in α-carotene, zeaxanthin, and β-cryptoxanthin, while lutein levels decreased significantly. In contrast, CAN2 was linked to genetic changes that led to a significant (*P*<0.0001) reduction in provitamin A, mainly due to lower (*P*<0.0001) β-carotene content and an increase (*P*<0.05) in lutein in hybrid kernels. Meanwhile, CAN3 promoted a significant (*P*<0.05 to *P*<0.0001) increase in provitamin A by enhancing accumulation of all carotenoids except for β-cryptoxanthin (**Table 2**). Consequently, the increases in provitamin A accumulation in hybrids seem to be primarily driven by significant rises in either β-cryptoxanthin and α-carotene or α-carotene and β-carotene, while also maintaining significantly higher levels of zeaxanthin. Interestingly, α-carotene demonstrated either a positive or negative impact on provitamin A accumulation in hybrids. A plot of CAN1 and CAN2 scores that incorporated the results of Ward’s clustering method, categorized the hybrids into four distinct groups with minimal overlap. GROUP-1 included 54 hybrids with positive CAN1 scores and a mix of positive and negative CAN2 scores, exhibiting moderate levels of lutein alongside high concentrations of other carotenoids, including provitamin A (**Table 3**). GROUP-2 comprises 26 hybrids showing positive and negative CAN1 scores combined with mainly positive CAN2 scores (**Figure 1**), characterized by moderate levels of zeaxanthin and low levels of other carotenoids, including provitamin A (**Table 3**). GROUP-3 consisted of 40 hybrids demonstrating a mix of positive and negative CAN1 scores coupled with mainly positive CAN2 scores (**Figure 1**), featuring moderate levels of lutein and zeaxanthin, low levels of β-cryptoxanthin and α-carotene, and high levels of β-carotene and provitamin A (**Table 3**). Finally, GROUP-4 included the remaining 12 hybrids, which exhibited mainly positive CAN1 and CAN2 scores (**Figure 1**), characterized by high concentrations of lutein and low levels of the remaining carotenoids, including provitamin A (**Table 3**).

**Table 2:** Correlation coefficients between hybrid BLUEs of carotenoids and canonical discriminant function scores.

Carotenoids	CAN1	CAN2	CAN3
Lutein	-0.39****	0.21*	0.81****
Zeaxanthin	0.52****	0.11	0.33****
β-Cryptoxanthin	0.96****	0.14	0.11
α-carotene	0.94****	-0.2	0.22*
β-carotene	0.17	-0.81****	0.49****
Provitamin A	0.51****	-0.65****	0.47****
Variance	0.72****	0.17****	0.12****

Simple correlation coefficients between hybrid BlUEs of carotenoids and CAN scores marked with * and **** are significantly different at *P*<0.05 and *P*<0.0001 levels, respectively.

**Table 3:** Means of carotenoids for groups of lines separated based on clustering of PCA axis scores for carotenoids excluding provitamin A.

Carotenoids	Number	Minimum	Maximum	Mean
GROUP-1				
Lutein (µg/g)	54	3.7	15.3	8.6 ± 0.28
Zeaxanthin (µg/g)	54	13.0	24.7	16.8 ± 0.36
β-cryptoxanthin (µg/g)	54	4.2	6.4	5.4 ± 0.07
σ-carotene (µg/g)	54	0.9	1.5	1.1 ± 0.02
β-carotene (µg/g)	54	5.0	8.8	6.3 ± 0.11
Provitamin A (µg/g)	54	7.9	12.4	9.6 ± 0.13
GROUP-2				
Lutein (µg/g)	26	3.6	9.5	6.7 ± 0.30
Zeaxanthin (µg/g)	26	6.9	22.0	13.4 ± 0.84
β-cryptoxanthin (µg/g)	26	3.0	6.0	4.3 ± 0.14
σ-carotene (µg/g)	26	0.6	1.0	0.8 ± 0.01
β-carotene (µg/g)	26	3.0	6.0	4.3 ± 0.12
Provitamin A (µg/g)	26	5.3	8.6	6.9 ± 0.17
GROUP-3				
Lutein (µg/g)	40	5.0	13.3	8.0 ± 0.28
Zeaxanthin (µg/g)	40	5.6	23.1	13.0 ± 0.60
β-cryptoxanthin (µg/g)	40	1.3	4.9	3.6 ± 0.13
σ-carotene (µg/g)	40	0.4	1.2	0.8 ± 0.03
β-carotene (µg/g)	40	4.8	9.6	6.9 ± 0.19
Provitamin A (µg/g)	40	6.7	12.1	9.3 ± 0.20
GROUP-4				
Lutein (µg/g)	12	8.9	16.4	12.3 ± 0.73
Zeaxanthin (µg/g)	12	4.2	21.7	11.0 ± 1.46
β-cryptoxanthin (µg/g)	12	0.5	2.8	1.8 ± 0.23
σ-carotene (µg/g)	12	0.0	0.8	0.3 ± 0.07
β-carotene (µg/g)	12	1.0	9.7	4.8 ± 0.92
Provitamin A (µg/g)	12	1.5	11.0	6.0 ± 1.01

### Genetic gain estimates for grain yield and other agronomic traits

The regional trials involving provitamin A-enriched hybrids were conducted across a wide range of rainfed field environments, with average grain yields varying from 443 kg ha^-1^ to 8929 kg ha^-1^. Among the 305 test environments, 232 had average grain yields exceeding 3000 kg ha^-1^. The repeatability values for the 252 test environments used to estimate realized genetic gains ranged from 0.20 to 0.96. A comparative assessment of grain yields for EPVA hybrids against COMY and RPVA hybrids, as well as FPYC cultivars, serves as an important indicator of the genetic improvements achieved over the years. Mean grain yields ranged from 2632 to 5669 kg ha^-1^ for EPVA hybrids, from 3861 to 5277 kg ha^-1^ for COMY hybrids, and from 3845 to 4754 kg ha^-1^ for RPVA hybrids (**Supplementary Table S4**). Realized genetic gain estimates revealed a significant (*P*<0.0001) annual yield improvement of 77 kg ha^-1^, corresponding to a relative yield gain of 1.88% (**Table 4**). Among other agronomic traits, only silking and plant aspect showed significant (*P*<0.05) negative genetic gains, while plant height exhibited a significant (*P*<0.001) positive genetic gain.

**Table 4:** Genetic gain estimates for agronomic traits of hybrids evaluated in collaborative regional trials for 12 years.

Agronomic traits	(%)	Slope
Grain yield (kg/ha)	1.88	76.880****
Anthesis (days)	-0.08	-0.047
Silking (days)	-0.09	-0.056*
Plant height (cm)	0.31	0.565***
Ear height (cm)	0.21	0.189
Husk cover (1-5)	0.17	0.003
Plant aspect (1-5)	-0.35	-0.009*
Ear aspect (1-5)	-0.35	-0.009

Slops marked with *, ***, and **** are significantly different at *P*<0.05, *P*<0.001, and *P*<0.0001 levels, respectively.

### Effect of changes in grain yield on other agronomic traits

Analysis of genetic gains for different agronomic traits and their impact on yield is required to gain an insight into the potential to develop hybrids with optimal trait combinations and enhanced productivity. The genetic correlations between anthesis and silking with plant and ear heights were significant and negative (**Supplementary Table S5**). Similarly, plant and ear heights exhibited significant and negative correlations with husk cover, plant aspect, and ear aspect scores. In contrast, anthesis and silking showed positive correlations with husk cover, plant aspect, and ear aspect scores. Positive and significant genetic correlations were also observed between husk cover and both plant aspect and ear aspect scores, as well as between plant aspect and ear aspect scores, anthesis and silking, and plant height and ear height. Furthermore, the genetic correlations of grain yield with plant height and ear height were significant and positive, while correlations with other traits were significant and negative. Additional analyses of changes in other agronomic traits using canonical discriminant analysis identified three significant discriminant functions (CAN1, CAN2, and CAN3), which accounted for 62%, 35%, and 3% of the total variation in agronomic traits measured in the hybrids, respectively (**Table 5**). In CAN1, significant (*P*<0.0001) increases in anthesis, silking, plant height, and ear height, along with desirable changes in husk cover, plant aspect, and ear aspect, were positively correlated with grain yield. Conversely, CAN2 showed a significant (*P*<0.001 to *P*<0.0001) negative correlation of grain yield with increases in anthesis, silking, plant aspect, and ear aspect, coupled with a decrease in plant height. Meanwhile, the significant (*P*<0.0001) positive correlations of plant height, ear height, and husk cover with CAN3 scores did not demonstrate a significant association with grain yield.

**Table 5:** Correlation coefficients between hybrid BLUEs of agronomic traits and canonical discriminant function scores.

Agronomic traits	CAN1	CAN2	CAN3
Anthesis	0.63****	0.69****	-0.02
Silking	0.62****	0.76****	-0.07
Plant height	0.64****	-0.30***	0.45****
Ear height	0.71****	-0.08	0.61****
Husk cover	-0.67****	0.13	0.44****
Plant aspect	-0.57****	0.70****	0.16
Ear aspect	-0.60****	0.59****	-0.15
Grain yield	0.45****	-0.62****	0.19
Variance	0.62****	0.35****	0.03**

Simple correlation coefficients between hybrid BlUEs of agronomic traits and CAN scores marked with *** and **** are significantly different at *P*<0.001, and *P*<0.0001 levels, respectively.

**Figure 2:**
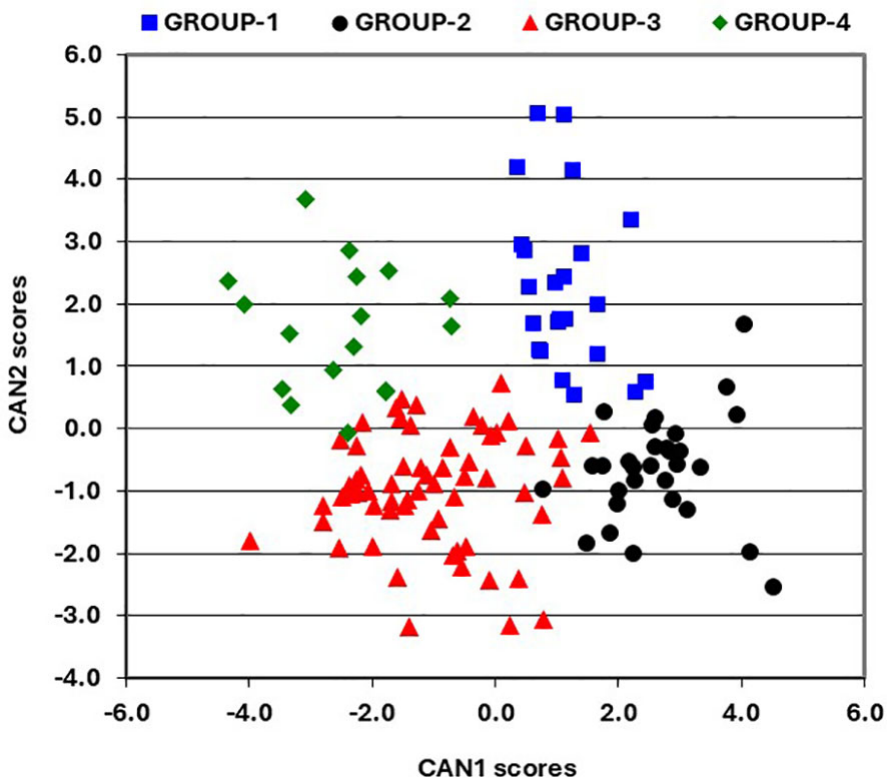
Scatter plot of the first and second canonical discriminant function scores for agronomic traits.

A scatter plot of CAN1 and CAN2 scores, incorporating the results of Ward’s clustering method, reveals four distinct groups of hybrids (**Figure 2**). GROUP-1 includes 23 hybrids with positive scores for both CAN1 and CAN2 (**Figure 2**), characterized by shorter plants, low ear placement, and lower mean grin yield (**Table 6**). GROUP-2 comprises 30 hybrids with positive CAN1 scores and negative CAN2 scores, recognized for their taller plants, high ear placement, and the highest mean grain yield. GROUP-3 consists of 62 hybrids exhibiting primarily negative scores for both CAN1 and CAN2 (**Figure 2**), featuring relatively shorter plants, low ear placement, and high mean grain yield. Finally, GROUP-4 includes the remaining 17 hybrids combining negative CAN1 scores with mainly positive CAN2 scores, noted for their shorter plants, low ear placement, and the lowest mean grain yield (**Table 6**).

**Table 6:** Means of agronomic traits for groups of lines separated based on clustering of PCA axis scores for agronomic traits excluding yield.

Agronomic traits	Number	Minimum	Maximum	Mean
GROUP-1				
Anthesis (days)	23	59	61	59 ± 0.15
Silking (days)	23	61	64	62 ± 0.15
Plant height (cm)	23	170	192	182 ± 1.02
Ear height (cm)	23	82	94	90 ± 0.66
Husk cover (1-5)	23	1.9	2.1	1.9 ± 0.01
Plant aspect (1-5)	23	2.4	2.9	2.6 ± 0.03
Ear aspect (1-5)	23	2.3	3.1	2.7 ± 0.04
Grain yield (kg/ha)	23	3024	4554	3839 ± 84
GROUP-2				
Anthesis (days)	30	58	60	59 ± 0.12
Silking (days)	30	60	63	61 ± 0.12
Plant height (cm)	30	186	198	192 ± 0.48
Ear height (cm)	30	92	105	96 ± 0.52
Husk cover (1-5)	30	1.7	2.0	1.9 ± 0.01
Plant aspect (1-5)	30	2.0	2.5	2.3 ± 0.02
Ear aspect (1-5)	30	2.0	2.6	2.3 ± 0.03
Grain yield (kg/ha)	30	3754	5690	4840 ± 83
GROUP-3				
Anthesis (days)	62	56	58	57 ± 0.09
Silking (days)	62	58	61	60 ± 0.09
Plant height (cm)	62	163	196	182 ± 0.86
Ear height (cm)	62	73	95	88 ± 0.57
Husk cover (1-5)	62	1.8	2.3	2.0 ± 0.01
Plant aspect (1-5)	62	2.2	2.7	2.4 ± 0.01
Ear aspect (1-5)	62	2.1	2.8	2.5 ± 0.02
Grain yield (kg/ha)	62	3050	5538	4461 ± 70
GROUP-4				
Anthesis (days)	17	57	59	58 ± 0.18
Silking (days)	17	59.4	62	60 ± 0.16
Plant height (cm)	17	163	187	178 ± 1.91
Ear height (cm)	17	78.4	94	87 ± 1.04
Husk cover (1-5)	17	1.8	2.6	2.2 ± 0.05
Plant aspect (1-5)	17	2.5	2.9	2.7 ± 0.03
Ear aspect (1-5)	17	2.5	3.1	2.8 ± 0.04
Grain yield (kg/ha)	17	2632	4430	3532 ± 147

### Effect of changes in carotenoid accumulation on agronomic traits

Assessing the impact of increased accumulation of carotenoids in hybrids on their agronomic traits is important to gain a deep understanding of the feasibility of developing hybrids with enhanced agronomic performance and elevated levels of beneficial carotenoids. The simple correlations between carotenoid concentrations and canonical discriminant function scores for agronomic traits are summarized in **Table 7**. All carotenoids, including provitamin A, exhibited significant (*P*<0.05 *to P*<0.0001) positive correlations with CAN1 scores for agronomic traits. This indicates that increases in the accumulation of all carotenoids, including provitamin A, were associated with late flowering time, taller plants, higher ear placement, good husk cover, desirable plant and ear aspect scores, and increased grain yield (**Tables 7**). Conversely, only three agronomic traits in CAN1, one trait in CAN2, and five traits in CAN3 showed significant (*P*<0.05 to *P*<0.0001) positive or negative correlations with the canonical discriminant function scores for carotenoids (**Table 8**). Notably, grain yield was not correlated with CAN1 and CAN3 scores but demonstrated a weak negative correlation (*P*<0.05) with CAN2 scores.

**Table 7:** Correlations of carotenoid concentrations with three canonical discriminant function (CAN1, CAN2, CAN3) scores for agronomic traits.

	Agronomic traits		
Carotenoids	CAN1 scores	CAN2 scores	CAN3 scores
Lutein (µg/g)	0.19*	0.08	0.12
Zeaxanthin (µg/g)	0.23**	0.14	0.16
β-cryptoxanthin (µg/g)	0.30***	0.16	-0.05
σ-carotene (µg/g)	0.42****	0.18*	-0.06
β-carotene (µg/g)	0.30**	0.11	0.02
Provitamin A (µg/g)	0.38****	0.14	0.01

Simple correlation coefficients between carotenoid content and CAN1, CAN2 and CAN3 scores of agronomic traits marked with *, **, ***, and **** are significantly different at *P*<0.05, *P*<0.01, *P*<0.001, and *P*<0.0001 levels, respectively.

**Table 8:** Correlations of agronomic traits with canonical discriminant function (CAN1, CAN2, CAN3) scores for carotenoids.

	Carotenoids		
Agronomic traits	CAN1 scores	CAN2 scores	CAN3 scores
Anthesis (days)	0.29***	-0.16	0.34****
Silking (days)	0.30***	-0.13	0.34****
Plant height (cm)	0.11	-0.23**	0.26**
Ear height (cm)	0.10	-0.12	0.36****
Husk cover (1-5)	-0.25**	0.07	-0.18*
Plant aspect (1-5)	-0.07	0.07	-0.04
Ear aspect (1-5)	-0.14	-0.07	-0.09
Grain yield (kg/ha)	-0.03	-0.20*	0.14

Simple correlation coefficients between agronomic traits and CAN1, CAN2 and CAN3 scores of carotenoids marked with *, **, ***, and **** are significantly different at *P*<0.05, *P*<0.01, *P*<0.001, and *P*<0.0001 levels, respectively.

### Hybrids combining elevated levels of provitamin A with high grain yields

Examining the feasibility of developing hybrids that combine high provitamin A content with acceptable or superior agronomic performance across a wide range of growing environments is required to commercialize and cultivate the hybrids in smallholder farmers’ fields. In this study, the top 30% of the EPHV hybrids evaluated over a minimum of two years exhibited provitamin A content ranging from 9.0 to 12.4 µg/g, with grain yields varying from 4035 and 5505 kg/ha (**Supplementary Tables S3, S5**). These hybrids were competitive with or better than the COMY and RPVA hybrids in their yield potential. As a group, the best 30% of the EPHV hybrids yielded 4% more than the COMY hybrids, 13% more than the RPVA hybrids, and 30% more than the FPYC cultivars. Furthermore, the average increase in provitamin A content for the best 30% EPVH hybrids was 124% compared to the COMYH, 6% compared to the RPVA hybrids, and 79% compared to FPYC cultivars.

## Discussion

For nearly two decades, maize breeders have focused on enhancing β-carotene accumulation while selecting desirable agronomic traits to develop and deliver provitamin A-enriched maize varieties in regions with high vitamin A deficiency (Pfeiffer and McClafferty, 2007). This breeding strategy has also been employed at IITA to create elite provitamin A-enriched hybrids, which have been shared with national agricultural institutes and private seed companies for performance testing across diverse growing conditions. Collaborative regional trials of these hybrids, which were conducted by partners for 12 years, were used to assess the effectiveness of this breeding approach in achieving genetic gains in both provitamin A content and grain yield. The significant annual increase in provitamin A content observed in this study was primarily driven by enhanced β-carotene accumulation, alongside a significant reduction in β-cryptoxanthin levels. These results indicate that visually selecting for orange-yellow kernel color and flint to semi-flint kernel texture, combined with selecting for carotenoid content measured via HPLC in parental lines, effectively directed metabolic flux to the β-branch of the carotenoid biosynthetic pathway while reducing the hydroxylation of β-carotene to β-cryptoxanthin and zeaxanthin (Wurtzel et al., 2012).

Enhancing accumulations of both carotenes and xanthophylls in hybrids is important to maximize the health benefits derived from maize. While the overall genetic gain in provitamin A content was mainly associated with increased β-carotene levels in the present study, the results from the canonical discriminant analysis revealed that hybrid groups followed different pathways to achieve high concentrations of provitamin A. The hybrids in the first group accumulated moderate to high concentrations of both provitamin A and other carotenoids, while those in the third group displayed moderate to high concentrations of lutein, zeaxanthin, and β-carotene, along with low levels of β-cryptoxanthin and α-carotene. Hybrids in the second and fourth groups displayed low levels of provitamin A carotenoids, with either low or high levels of lutein and zeaxanthin, leading to reduced provitamin A content. The observed variations in carotenoid profiles and content in hybrids may thus originate from crosses of diverse parental lines possessing different favorable alleles of both known and unknown genes. These genes that originated from diverse source germplasm could regulate variable carotenoid synthesis and substrate channeling to the α- and β-branches of the carotenoid biosynthetic pathway (Sun et al., 2018; Kandianis et al., 2013; Menkir et al., 2017). Consequently, our breeding strategy was effective in assembling numerous favorable alleles in elite maize inbred lines, leading to hybrids with distinct carotenoid composition and content.

Given the significant genetic gains achieved in β-carotene content in our study, adopting a breeding strategy that also targets both carotenes and xanthophylls is important to further enhance provitamin A and other beneficial carotenoids for human health. Increased efforts should then focus on boosting β-cryptoxanthin levels in maize kernels, as the present study recorded a significant negative genetic gain in its content. β-cryptoxanthin is more bioavailable than β-carotene, making it an effective source of retinol for humans (Burri et al., 2011; Burt et al., 2011; Burri et al., 2016). Additionally, β-cryptoxanthin is converted into vitamin A more efficiently than β-carotene, facilitating better absorption in the human body (Johnson, 2002; Tanumihardjo, 2013; Johnson and Russell, 2016). Beyond its role in vitamin A supply, the strong antioxidant properties of β-cryptoxanthin are associated with a reduced risk of chronic diseases, including cancer and osteoporosis (Johnson, 2002; Burri et al., 2016; Ma and Liu, 2018). Research indicates that β-cryptoxanthin is more stable than β-carotene in grains, flour, and under various storage and processing conditions (Trono, 2019). Consequently, selection for regulatory genes modulating substrate synthesis upstream in the biosynthetic pathway and direct flux to both the α- and β-branches can have a significant impact on increasing both carotene and xanthophylls to much higher levels. The yellow-orange color in maize endosperm imparted by these carotenoids coupled with desirable kernel texture can thus be used to select early-generation lines through visual assessment or chromameter values (Menkir et al., 2017; Venado et al., 2017). Consuming maize enriched with high concentrations of both carotene and xanthophyll may thus have a synergistic effect on antioxidant activities, promoting better human health (Boileau and Clinton, 2008).

Assessing the impact of the current provitamin A enrichment strategy on yield gains across different growing conditions is essential to generate hybrids attractive to seed companies for commercialization in tropical lowland savannas, where vitamin A deficiency is widespread. The annual yield gain of 77 kg ha−1 achieved over a 12-year period is comparable to the 66 to 143 kg ha−1 year−1 yield increases reported in other genetic gain studies of late-maturing hybrids (Haegele et al., 2013; Smith et al., 2014; Chen et al., 2017; DeBruin et al., 2017; Fernández et al., 2022; 61). Selecting for high provitamin A content and desirable agronomic traits in parental lines has therefore successfully imparted increased yield gains in hybrids across diverse environments with varying production potentials and management practices typical of smallholder farmers’ fields. Further assessment of changes in agronomic traits through canonical discriminant analysis found that delays in flowering, increased height, and favorable changes in husk cover and other traits were linked to enhanced grain yield. Two hybrid groups with contrasting plant heights and ear placements had high average grain yields, whereas the other two hybrid groups with shorter plants and lower ear placement produced lower average grain yields. These findings highlight that breeding for enhanced carotenoid content can be accompanied by superior agronomic performance.

As maize continues to serve as a major source of food and nutrition over the next 20–30 years, the greatest potential impact may arise from high yielding varieties that provide essential vitamins and minerals Virk et al., 2021). In the present study, we identified 23 provitamin A-enriched hybrids that accumulated 54 to 95% more provitamin A and produced 10 to 30% higher grain yields compared to the best commercial hybrid (COMYH129) having high provitamin A, making them suitable for commercialization by indigenous seed companies. Amongst all the provitamin A-enriched hybrids included in the collaborative regional trials, 18 have been released for commercialization in Ghana, Mali, and Nigeria. Additionally, IITA granted exclusive licenses to four seed companies to incentivize investments in the production and marketing of five provitamin A-enriched hybrids in Burkina Faso, Ghana, Mali, and Nigeria. These results demonstrate the effectiveness of our breeding strategy in assembling desirable traits and allelic combinations, leading to high provitamin A content and superior agronomic performance of the hybrids across diverse growing conditions (Menkir et al., 2021). Additionally, the positive correlations between carotenoid content and CAN1 scores for agronomic traits underscore the feasibility in achieving significant productivity gains alongside increased concentrations of provitamin A and other beneficial carotenoids for sustainable food and nutritional security. However, the achievements made so far in provitamin A content and yield potential are insufficient to meet the projected increased needs of the vulnerable consumers in sub-Saharan Africa. Further enhancements in productivity growth and increases in accumulating provitamin A and other beneficial carotenoids may be achieved by continually infusing novel alleles from diverse temperate yellow maize germplasm that received significant research investment for decades in industrialized countries. This approach will facilitate the stacking of novel alleles associated with genes regulating the increased synthesis and accumulation of multiple carotenoids and superior agronomic attributes to develop more nutritious and appealing maize breeding pipelines.

## Summary

The biofortification strategy successfully enhanced both provitamin A levels and grain yield in tropical maize hybrids through strategic integration of diverse germplasm, multistage visual selection for desirable agronomic and adaptive traits, and measuring carotenoids of advanced maize inbred lines using HPLC. While these hybrids address vitamin A deficiency and benefit both rural and urban consumers, meeting the full daily vitamin A requirement requires optimizing accumulation of provitamin A carotenoids, reducing degradation and improving sequestration. Systematic integration of molecular breeding tools targeting genes involved in carotenoid biosynthesis with modern conventional breeding methods will accelerate genetic gains in accumulating beneficial carotenoids and improving agronomic performance.

The long-term breeding strategy uses tropical-adapted maize inbred lines representing two heterotic groups with complementary regulatory gene networks for carotenoid biosynthesis to develop synthetic populations for improvement through rapid-cycle genomic selection. The resulting divergent lines derived from the two synthetics can then be used as parents to optimize accumulation of beneficial carotenoids and enhance agronomic performance in tropical maize hybrids. Divergent lines derived from these synthetic populations can then serve as parents to simultaneously optimize beneficial carotenoid to human health and boost agronomic performance in tropical maize hybrids.

## Data Availability

The original contributions presented in the study are included in the article/**Supplementary Material**. Further inquiries can be directed to the corresponding author.
