# Supplementary material for: Concurrent enhancement of provitamin A and yield in tropical maize hybrids
Source: Front Plant Sci. 2025 Jul 17;16:1611495. doi: 10.3389/fpls.2025.1611495 (PMC12310686; doi:10.3389/fpls.2025.1611495)
Supplement: Supplementary file 1 [file Table1.docx]

Table S1. List of provitamin A enriched hybrids and commercial and released checks evaluated over 12-years across diverse test environments.

| Entry | Hybrid | Hybrid name |
| --- | --- | --- |
| 1 | EPVA002 | A0807-70 |
| 2 | EPVA003 | A0902-19 |
| 3 | EPVA004 | A0902-7 |
| 4 | EPVA005 | A0905-23 |
| 5 | EPVA006 | A0905-34 |
| 6 | EPVA007 | A0905-35 |
| 7 | EPVA008 | A1312-12 |
| 8 | EPVA009 | A1312-4 |
| 9 | EPVA010 | A1702-28 |
| 10 | EPVA011 | A1702-49 |
| 11 | EPVA012 | A1702-53 |
| 12 | EPVA013 | A1703-15 |
| 13 | EPVA014 | A1705-2 |
| 14 | EPVA015 | A1706-2 |
| 15 | EPVA016 | A1736-12 |
| 16 | EPVA017 | A1736-13 |
| 17 | EPVA018 | A1736-15 |
| 18 | EPVA019 | A1736-2 |
| 19 | EPVA020 | A1736-6 |
| 20 | EPVA021 | A1736-9 |
| 21 | EPVA022 | A1801-43 |
| 22 | EPVA023 | A1801-44 |
| 23 | EPVA024 | A1802-12 |
| 24 | EPVA025 | A1802-16 |
| 25 | EPVA026 | A1802-4 |
| 26 | EPVA027 | A1804-14 |
| 27 | EPVA028 | A1804-15 |
| 28 | EPVA029 | A1804-66 |
| 29 | EPVA030 | A1804-67 |
| 30 | EPVA035 | LY0614-11 |
| 31 | EPVA036 | LY0704-8 |
| 32 | EPVA037 | LY0902-1 |
| 33 | EPVA038 | LY0902-12 |
| 34 | EPVA039 | LY0902-18 |
| 35 | EPVA040 | LY0902-19 |
| 36 | EPVA041 | LY0902-24 |
| 37 | EPVA042 | LY0902-27 |
| 38 | EPVA043 | LY0902-33 |
| 39 | EPVA044 | LY0902-34 |
| 40 | EPVA045 | LY0902-38 |
| 41 | EPVA046 | LY0902-40 |
| 42 | EPVA047 | LY0905-34 |
| 43 | EPVA048 | LY0905-35 |
| 44 | EPVA049 | LY0906-19 |
| 45 | EPVA050 | LY0906-8 |
| 46 | EPVA051 | LY1001-10 |
| 47 | EPVA052 | LY1001-14 |
| 48 | EPVA053 | LY1001-18 |
| 49 | EPVA054 | LY1001-21 |
| 50 | EPVA055 | LY1001-22 |
| 51 | EPVA056 | LY1001-23 |
| 52 | EPVA057 | LY1203-43 |
| 53 | EPVA058 | LY1212-14 |
| 54 | EPVA059 | LY1302-9 |
| 55 | EPVA060 | LY1303-18 |
| 56 | EPVA061 | LY1303-5 |
| 57 | EPVA062 | LY1312-11 |
| 58 | EPVA063 | LY1312-12 |
| 59 | EPVA064 | LY1312-20 |
| 60 | EPVA065 | LY1312-25 |
| 61 | EPVA066 | LY1312-28 |
| 62 | EPVA067 | LY1312-30 |
| 63 | EPVA068 | LY1312-4 |
| 64 | EPVA069 | LY1312-9 |
| 65 | EPVA070 | LY1409-12 |
| 66 | EPVA071 | LY1409-14 |
| 67 | EPVA072 | LY1409-21 |
| 68 | EPVA073 | LY1409-61 |
| 69 | EPVA074 | LY1409-62 |
| 70 | EPVA075 | LY1501-1 |
| 71 | EPVA076 | LY1501-2 |
| 72 | EPVA077 | LY1501-3 |
| 73 | EPVA078 | LY1501-4 |
| 74 | EPVA079 | LY1501-5 |
| 75 | EPVA080 | LY1501-6 |
| 76 | EPVA081 | LY1501-7 |
| 77 | EPVA082 | LY1501-8 |
| 78 | EPVA083 | LY1501-9 |
| 79 | EPVA084 | LY1601 |
| 80 | EPVA085 | LY1605 |
| 81 | EPVA086 | LY1606 |
| 82 | EPVA087 | LY1607 |
| 83 | EPVA088 | LY1608 |
| 84 | EPVA089 | LY1609 |
| 85 | EPVA090 | LY1610 |
| 86 | EPVA091 | LY1611 |
| 87 | EPVA092 | LY1612 |
| 88 | EPVA093 | LY1613 |
| 89 | EPVA094 | LY1614 |
| 90 | EPVA095 | LY1615 |
| 91 | EPVA096 | LY1901-11 |
| 92 | EPVA097 | LY1901-12 |
| 93 | EPVA098 | LY1901-13 |
| 94 | EPVA099 | LY1901-14 |
| 95 | EPVA100 | LY1901-15 |
| 96 | EPVA101 | LY1901-16 |
| 97 | EPVA102 | LY1901-17 |
| 98 | EPVA103 | LY1901-18 |
| 99 | EPVA104 | LY1901-19 |
| 100 | EPVA105 | LY1901-20 |
| 101 | EPVA106 | LY1901-21 |
| 102 | EPVA107 | LY1901-22 |
| 103 | EPVA108 | LY1901-23 |
| 104 | EPVA109 | LY1901-24 |
| 105 | EPVA110 | LY1901-25 |
| 106 | EPVA111 | LY1913-16 |
| 107 | EPVA112 | LY1913-23 |
| 108 | EPVA113 | LY1913-3 |
| 109 | EPVA114 | LY1914-14 |
| 110 | EPVA115 | LY20001-1 |
| 111 | EPVA116 | LY20001-10 |
| 112 | EPVA117 | LY20001-11 |
| 113 | EPVA118 | LY20001-12 |
| 114 | EPVA119 | LY20001-13 |
| 115 | EPVA120 | LY20001-2 |
| 116 | EPVA121 | LY20001-3 |
| 117 | EPVA122 | LY20001-4 |
| 118 | EPVA123 | LY20001-5 |
| 119 | EPVA124 | LY20001-6 |
| 120 | EPVA125 | LY20001-7 |
| 121 | EPVA126 | LY20001-8 |
| 122 | EPVA127 | LY20001-9 |
| 123 | EPVA128 | M1124-31 |
| 124 | EPVA132 | LY0614-8 |
| 125 | COMYH001 | COMYH001 |
| 126 | COMYH129 | COMYH129 |
| 127 | COMYH130 | COMYH130 |
| 128 | COMYH031 | COMYH031 |
| 129 | RPVA032 | Ife Hybrid-3 |
| 130 | RPVA033 | Ife Hybrid-4 |
| 131 | RPVA131 | SAMMAZ 44 |
| 132 | FPYC034 | Local check |

Table S2. Hybrid means (BLUEs) in µg/g for provitamin A (PVA)and other carotenoids recorded over 12 years across locations

| Hybrid | Number of years of testing | Lutein | Zeaxanthin | β-Cryptoxanthin | α-carotene | β-carotene | PVA |
| --- | --- | --- | --- | --- | --- | --- | --- |
| EPVA002 | 2 | 8.5 | 14.4 | 4.9 | 0.9 | 4.4 | 7.3 |
| EPVA003 | 1 | 6.6 | 12.2 | 4.9 | 0.9 | 4.9 | 7.8 |
| EPVA004 | 2 | 5.4 | 14.6 | 5.0 | 0.8 | 4.5 | 7.4 |
| EPVA005 | 3 | 8.7 | 13.9 | 3.6 | 0.9 | 5.4 | 7.7 |
| EPVA006 | 3 | 11.0 | 13.9 | 4.2 | 1.0 | 6.2 | 8.7 |
| EPVA007 | 3 | 8.0 | 20.4 | 6.3 | 1.1 | 6.1 | 9.8 |
| EPVA008 | 2 | 8.7 | 13.4 | 4.8 | 0.9 | 7.0 | 10.0 |
| EPVA009 | 1 | 8.0 | 18.6 | 5.6 | 1.2 | 6.6 | 10.0 |
| EPVA010 | 3 | 7.9 | 15.2 | 5.6 | 1.1 | 5.8 | 9.2 |
| EPVA011 | 2 | 7.3 | 20.2 | 6.2 | 1.3 | 6.6 | 10.3 |
| EPVA012 | 2 | 8.2 | 19.3 | 6.2 | 1.4 | 6.9 | 10.7 |
| EPVA013 | 1 | 6.5 | 7.8 | 4.5 | 0.9 | 5.5 | 8.3 |
| EPVA014 | 1 | 11.6 | 14.3 | 4.2 | 1.0 | 5.1 | 7.9 |
| EPVA015 | 4 | 6.9 | 14.2 | 3.3 | 0.7 | 7.8 | 10.1 |
| EPVA016 | 3 | 6.8 | 17.5 | 5.6 | 1.1 | 5.7 | 9.1 |
| EPVA017 | 4 | 5.5 | 19.4 | 5.6 | 1.1 | 5.8 | 9.2 |
| EPVA018 | 1 | 8.5 | 14.5 | 4.8 | 1.0 | 5.0 | 8.0 |
| EPVA019 | 1 | 6.7 | 8.8 | 2.7 | 0.5 | 7.6 | 9.4 |
| EPVA020 | 4 | 5.8 | 9.4 | 4.0 | 0.8 | 8.8 | 11.5 |
| EPVA021 | 1 | 15.3 | 15.0 | 5.1 | 1.2 | 5.0 | 8.1 |
| EPVA022 | 1 | 6.9 | 11.0 | 4.2 | 0.8 | 6.9 | 9.5 |
| EPVA023 | 1 | 13.3 | 13.4 | 3.6 | 0.8 | 7.2 | 9.6 |
| EPVA024 | 1 | 6.2 | 17.9 | 4.4 | 1.0 | 6.5 | 9.3 |
| EPVA025 | 1 | 9.7 | 15.5 | 3.5 | 0.7 | 5.9 | 8.4 |
| EPVA026 | 3 | 6.3 | 13.1 | 4.8 | 1.0 | 7.0 | 10.0 |
| EPVA027 | 3 | 6.9 | 10.9 | 4.8 | 0.9 | 7.4 | 10.4 |
| EPVA028 | 3 | 6.5 | 10.8 | 4.9 | 1.0 | 7.0 | 10.1 |
| EPVA029 | 2 | 6.3 | 13.8 | 3.4 | 0.8 | 8.2 | 10.5 |
| EPVA030 | 3 | 5.0 | 12.9 | 4.6 | 0.9 | 6.6 | 9.4 |
| EPVA035 | 3 | 9.5 | 7.6 | 3.3 | 0.8 | 4.1 | 6.1 |
| EPVA036 | 1 | 7.9 | 12.0 | 3.0 | 0.7 | 3.5 | 5.3 |
| EPVA037 | 1 | 7.4 | 12.6 | 4.7 | 0.8 | 4.9 | 7.8 |
| EPVA038 | 2 | 5.1 | 10.8 | 5.4 | 0.8 | 4.1 | 7.4 |
| EPVA039 | 2 | 7.5 | 13.2 | 4.4 | 0.9 | 4.1 | 6.7 |
| EPVA040 | 4 | 5.6 | 11.3 | 4.5 | 0.8 | 4.4 | 7.0 |
| EPVA041 | 1 | 6.8 | 16.1 | 5.0 | 0.9 | 4.5 | 7.5 |
| EPVA042 | 1 | 3.6 | 19.2 | 4.7 | 0.8 | 4.1 | 6.9 |
| EPVA043 | 1 | 6.6 | 18.7 | 5.8 | 1.0 | 5.4 | 8.9 |
| EPVA044 | 1 | 4.7 | 6.9 | 3.9 | 0.8 | 4.0 | 6.3 |
| EPVA045 | 1 | 4.3 | 12.3 | 3.9 | 0.8 | 3.9 | 6.2 |
| EPVA046 | 1 | 8.6 | 13.1 | 4.4 | 0.8 | 4.5 | 7.1 |
| EPVA047 | 1 | 8.5 | 15.4 | 5.3 | 1.3 | 6.9 | 10.4 |
| EPVA048 | 1 | 9.3 | 18.3 | 6.4 | 1.4 | 6.5 | 10.6 |
| EPVA049 | 1 | 5.5 | 20.6 | 4.1 | 0.8 | 3.9 | 6.3 |
| EPVA050 | 2 | 6.3 | 16.4 | 5.9 | 0.8 | 4.3 | 7.7 |
| EPVA051 | 5 | 6.4 | 12.0 | 4.8 | 1.1 | 6.9 | 9.8 |
| EPVA052 | 4 | 9.1 | 16.4 | 5.3 | 1.0 | 5.8 | 8.9 |
| EPVA053 | 9 | 9.1 | 14.4 | 4.8 | 1.0 | 6.4 | 9.3 |
| EPVA054 | 4 | 8.2 | 14.2 | 5.2 | 1.1 | 6.2 | 9.6 |
| EPVA055 | 6 | 9.6 | 15.2 | 5.0 | 1.0 | 6.1 | 9.0 |
| EPVA056 | 10 | 9.3 | 15.4 | 4.5 | 0.9 | 5.7 | 8.5 |
| EPVA057 | 2 | 6.8 | 13.2 | 3.8 | 0.8 | 7.2 | 9.4 |
| EPVA058 | 1 | 6.6 | 12.1 | 4.6 | 0.8 | 4.7 | 7.5 |
| EPVA059 | 6 | 8.1 | 17.4 | 5.0 | 1.0 | 5.6 | 8.7 |
| EPVA060 | 3 | 8.2 | 15.1 | 5.2 | 1.0 | 6.1 | 9.1 |
| EPVA061 | 2 | 7.5 | 12.2 | 4.1 | 0.9 | 5.7 | 8.2 |
| EPVA062 | 7 | 9.1 | 15.3 | 4.9 | 1.0 | 5.3 | 8.3 |
| EPVA063 | 7 | 8.1 | 15.2 | 5.4 | 1.1 | 5.7 | 9.1 |
| EPVA064 | 3 | 9.9 | 12.5 | 3.7 | 1.0 | 6.6 | 9.0 |
| EPVA065 | 1 | 7.9 | 12.2 | 3.4 | 0.6 | 6.7 | 8.8 |
| EPVA066 | 2 | 9.5 | 13.4 | 6.1 | 1.2 | 7.0 | 11.3 |
| EPVA067 | 2 | 8.1 | 9.6 | 3.9 | 0.9 | 5.9 | 8.2 |
| EPVA068 | 6 | 8.9 | 18.4 | 5.0 | 1.1 | 6.3 | 9.4 |
| EPVA069 | 2 | 8.1 | 18.2 | 4.9 | 1.0 | 5.7 | 9.1 |
| EPVA070 | 1 | 8.4 | 14.6 | 5.0 | 1.0 | 7.6 | 10.8 |
| EPVA071 | 6 | 7.7 | 17.0 | 4.4 | 0.9 | 6.4 | 9.1 |
| EPVA072 | 9 | 10.4 | 15.3 | 5.0 | 1.0 | 6.1 | 9.2 |
| EPVA073 | 4 | 7.9 | 16.1 | 4.9 | 1.0 | 6.6 | 9.6 |
| EPVA074 | 1 | 7.8 | 12.4 | 3.8 | 0.7 | 6.3 | 8.8 |
| EPVA075 | 6 | 11.0 | 14.5 | 4.8 | 1.0 | 6.3 | 9.3 |
| EPVA076 | 1 | 11.6 | 16.7 | 5.6 | 1.2 | 6.6 | 10.0 |
| EPVA077 | 4 | 10.6 | 20.6 | 5.9 | 1.3 | 6.6 | 10.2 |
| EPVA078 | 2 | 8.8 | 20.2 | 5.6 | 1.4 | 7.2 | 10.6 |
| EPVA079 | 6 | 8.5 | 14.6 | 5.0 | 1.0 | 6.6 | 9.7 |
| EPVA080 | 6 | 7.0 | 17.8 | 5.3 | 1.1 | 7.7 | 11.0 |
| EPVA081 | 7 | 9.1 | 17.4 | 5.6 | 1.2 | 6.1 | 9.6 |
| EPVA082 | 6 | 8.3 | 17.0 | 5.4 | 1.1 | 6.9 | 10.2 |
| EPVA083 | 6 | 7.5 | 16.1 | 5.7 | 1.2 | 7.1 | 10.6 |
| EPVA084 | 1 | 8.1 | 14.7 | 5.2 | 1.1 | 6.1 | 9.2 |
| EPVA085 | 1 | 9.3 | 12.2 | 4.1 | 0.8 | 4.2 | 6.8 |
| EPVA086 | 3 | 10.0 | 17.4 | 5.5 | 1.1 | 5.5 | 8.8 |
| EPVA087 | 1 | 6.6 | 17.1 | 6.0 | 1.1 | 5.8 | 9.5 |
| EPVA088 | 2 | 12.4 | 24.7 | 6.2 | 1.2 | 6.7 | 10.4 |
| EPVA089 | 2 | 8.4 | 14.2 | 5.0 | 1.0 | 5.1 | 8.1 |
| EPVA090 | 2 | 8.2 | 17.5 | 3.2 | 0.6 | 5.6 | 7.5 |
| EPVA091 | 1 | 8.9 | 4.2 | 0.5 | 0.0 | 2.6 | 2.8 |
| EPVA092 | 1 | 10.5 | 5.5 | 0.6 | 0.0 | 1.2 | 1.5 |
| EPVA093 | 1 | 10.5 | 5.8 | 0.7 | 0.0 | 2.1 | 2.4 |
| EPVA094 | 1 | 12.0 | 11.6 | 1.8 | 0.3 | 3.0 | 4.1 |
| EPVA095 | 1 | 5.1 | 8.6 | 2.9 | 0.4 | 5.7 | 7.6 |
| EPVA096 | 1 | 3.7 | 16.9 | 6.2 | 1.5 | 8.1 | 12.0 |
| EPVA097 | 2 | 14.2 | 8.7 | 1.9 | 0.5 | 7.1 | 8.7 |
| EPVA098 | 1 | 15.1 | 13.7 | 2.1 | 0.4 | 9.7 | 11.0 |
| EPVA099 | 2 | 9.0 | 17.2 | 3.8 | 1.0 | 7.2 | 9.9 |
| EPVA100 | 1 | 7.6 | 21.0 | 4.3 | 1.2 | 6.1 | 9.0 |
| EPVA101 | 2 | 8.0 | 15.4 | 3.6 | 1.0 | 9.6 | 12.1 |
| EPVA102 | 1 | 15.0 | 10.5 | 2.4 | 0.4 | 6.8 | 8.3 |
| EPVA103 | 2 | 10.0 | 15.5 | 3.1 | 0.9 | 8.9 | 11.1 |
| EPVA104 | 2 | 14.3 | 14.0 | 2.1 | 0.8 | 8.2 | 9.9 |
| EPVA105 | 2 | 6.7 | 20.7 | 3.8 | 0.8 | 4.9 | 7.3 |
| EPVA106 | 2 | 5.9 | 18.2 | 5.8 | 1.2 | 8.8 | 12.4 |
| EPVA107 | 1 | 16.4 | 8.8 | 1.4 | 0.3 | 8.9 | 9.9 |
| EPVA108 | 2 | 7.7 | 15.4 | 3.1 | 0.8 | 8.5 | 10.5 |
| EPVA109 | 2 | 11.7 | 15.0 | 3.3 | 1.0 | 6.9 | 9.2 |
| EPVA110 | 1 | 11.9 | 15.5 | 2.7 | 0.6 | 6.3 | 8.1 |
| EPVA111 | 2 | 8.1 | 23.1 | 3.5 | 0.9 | 8.6 | 10.9 |
| EPVA112 | 1 | 6.2 | 12.7 | 2.7 | 0.5 | 6.9 | 8.7 |
| EPVA113 | 1 | 10.3 | 10.7 | 2.7 | 0.4 | 5.2 | 7.1 |
| EPVA114 | 2 | 9.1 | 9.8 | 4.5 | 1.0 | 6.3 | 9.2 |
| EPVA115 | 1 | 10.6 | 15.5 | 3.6 | 0.8 | 4.8 | 7.1 |
| EPVA116 | 1 | 7.4 | 6.7 | 1.3 | 0.6 | 5.9 | 7.0 |
| EPVA117 | 1 | 6.2 | 7.1 | 4.0 | 0.8 | 6.0 | 8.6 |
| EPVA118 | 1 | 7.8 | 10.1 | 4.2 | 0.8 | 5.7 | 8.3 |
| EPVA119 | 1 | 8.5 | 13.5 | 2.5 | 0.9 | 5.0 | 6.7 |
| EPVA120 | 1 | 7.2 | 14.6 | 5.9 | 1.1 | 5.4 | 9.1 |
| EPVA121 | 1 | 8.5 | 5.6 | 1.9 | 0.8 | 7.8 | 9.4 |
| EPVA122 | 1 | 7.0 | 22.0 | 4.8 | 1.0 | 3.0 | 5.9 |
| EPVA123 | 1 | 7.6 | 6.0 | 2.1 | 0.8 | 9.2 | 10.8 |
| EPVA124 | 1 | 8.2 | 8.1 | 4.3 | 1.0 | 8.5 | 11.5 |
| EPVA125 | 1 | 5.4 | 23.4 | 5.5 | 1.0 | 5.0 | 8.3 |
| EPVA126 | 1 | 5.9 | 24.0 | 5.4 | 1.0 | 7.0 | 10.4 |
| EPVA127 | 1 | 4.6 | 15.6 | 5.9 | 1.1 | 6.7 | 10.1 |
| EPVA128 | 3 | 8.7 | 15.1 | 4.7 | 1.0 | 5.6 | 8.5 |
| EPVA132 | 2 | 8.6 | 7.1 | 3.3 | 0.8 | 3.9 | 5.9 |
| COMYH001 | 1 | 10.3 | 17.1 | 1.9 | 0.4 | 1.0 | 2.5 |
| COMYH129 | 1 | 5.0 | 13.5 | 3.3 | 0.8 | 3.7 | 5.9 |
| COMYH130 | 9 | 7.3 | 18.1 | 3.8 | 0.7 | 3.7 | 5.9 |
| COMYH031 | 1 | 10.3 | 21.7 | 2.8 | 0.4 | 1.9 | 3.6 |
| RPVA032 | 10 | 7.1 | 16.6 | 5.8 | 1.1 | 5.7 | 9.3 |
| RPVA033 | 10 | 11.2 | 13.0 | 4.4 | 1.1 | 6.2 | 8.9 |
| RPVA131 | 2 | 11.7 | 19.5 | 5.1 | 1.2 | 7.0 | 10.1 |
| FPYC034 | 9 | 7.1 | 14.7 | 3.4 | 0.6 | 3.5 | 5.6 |

Table S3. Repeatability values estimated for 62 environments.

| Environment | Lutein | Zeaxanthin | β-Cryptoxanthin | α-carotene | β-carotene | Provitamin A |
| --- | --- | --- | --- | --- | --- | --- |
| 1 | 0.83 | 0.89 | 0.90 | 0.94 | 0.92 | 0.95 |
| 2 | 0.00 | 0.07 | 0.56 | 0.00 | 0.00 | 0.82 |
| 3 | 0.00 | 0.95 | 0.66 | 0.87 | 0.77 | 0.92 |
| 4 | 0.43 | 0.77 | 0.53 | 0.61 | 0.55 | 0.83 |
| 5 | 0.33 | 0.83 | 0.53 | 0.83 | 0.88 | 0.76 |
| 6 | 0.67 | 0.94 | 0.64 | 0.91 | 0.91 | 0.79 |
| 7 | 0.79 | 0.89 | 0.82 | 0.94 | 0.95 | 0.84 |
| 8 | 0.85 | 0.76 | 0.41 | 0.82 | 0.83 | 0.80 |
| 9 | 0.72 | 0.83 | 0.91 | 0.84 | 0.82 | 0.96 |
| 10 | 0.75 | 0.68 | 0.89 | 0.65 | 0.69 | 0.85 |
| 11 | 0.08 | 0.97 | 0.74 | 0.90 | 0.95 | 0.95 |
| 12 | 0.93 | 0.91 | 0.84 | 0.97 | 0.94 | 0.94 |
| 13 | 0.91 | 0.94 | 0.93 | 0.98 | 0.97 | 0.84 |
| 14 | 0.50 | 0.00 | 0.52 | 0.31 | 0.34 | 0.32 |
| 15 | 0.00 | 0.00 | 0.15 | 0.00 | 0.00 | 0.67 |
| 16 | 0.92 | 0.95 | 0.89 | 0.98 | 0.98 | 0.85 |
| 17 | 0.92 | 0.86 | 0.92 | 0.92 | 0.92 | 0.45 |
| 18 | 0.00 | 0.70 | 0.53 | 0.21 | 0.21 | 0.57 |
| 19 | 0.33 | 0.69 | 0.30 | 0.44 | 0.16 | 0.14 |
| 20 | 0.57 | 0.81 | 0.66 | 0.91 | 0.92 | 0.79 |
| 21 | 0.14 | 0.00 | 0.00 | 0.13 | 0.13 | 0.00 |
| 22 | 0.72 | 0.59 | 0.92 | 0.80 | 0.84 | 0.77 |
| 23 | 0.80 | 0.83 | 0.87 | 0.83 | 0.91 | 0.89 |
| 24 | 0.56 | 0.88 | 0.77 | 0.96 | 0.93 | 0.36 |
| 25 | 0.42 | 0.55 | 0.71 | 0.21 | 0.10 | 0.63 |
| 26 |  | 0.84 | 0.82 | 0.82 | 0.78 | 0.86 |
| 27 |  | 0.88 | 0.73 | 0.73 | 0.56 | 0.81 |
| 28 | 0.45 | 0.59 | 0.57 | 0.65 | 0.62 | 0.70 |
| 29 | 0.25 | 0.75 | 0.71 | 0.70 | 0.64 | 0.87 |
| 30 | 0.78 | 0.78 | 0.43 | 0.35 | 0.32 | 0.55 |
| 31 | 0.78 | 0.76 | 0.68 | 0.81 | 0.76 | 0.87 |
| 32 | 0.00 | 0.37 | 0.32 | 0.52 | 0.63 | 0.20 |
| 33 | 0.42 | 0.36 | 0.00 | 0.00 | 0.00 | 0.10 |
| 34 | 0.17 | 0.48 | 0.83 | 0.57 | 0.85 | 0.46 |
| 35 | 0.80 | 0.72 | 0.57 | 0.54 | 0.57 | 0.85 |
| 36 | 0.76 | 0.79 | 0.00 | 0.68 | 0.65 | 0.36 |
| 37 | 0.75 | 0.76 | 0.66 | 0.82 | 0.78 | 0.70 |
| 38 | 0.90 | 0.90 | 0.41 | 0.85 | 0.76 | 0.79 |
| 39 | 0.94 | 0.95 | 0.59 | 0.90 | 0.82 | 0.79 |
| 40 | 0.81 | 0.82 | 0.33 | 0.84 | 0.82 | 0.57 |
| 41 | 0.90 | 0.92 | 0.55 | 0.89 | 0.81 | 0.76 |
| 42 | 0.89 | 0.85 | 0.72 | 0.90 | 0.88 | 0.46 |
| 43 | 0.33 | 0.75 | 0.71 | 0.82 | 0.80 | 0.84 |
| 44 | 0.62 | 0.81 | 0.72 | 0.82 | 0.87 | 0.62 |
| 45 | 0.07 | 0.66 | 0.26 | 0.70 | 0.68 | 0.66 |
| 46 | 0.51 | 0.64 | 0.47 | 0.61 | 0.70 | 0.67 |
| 47 | 0.58 | 0.68 | 0.71 | 0.79 | 0.88 | 0.74 |
| 48 | 0.86 | 0.88 | 0.69 | 0.79 | 0.78 | 0.87 |
| 49 | 0.82 | 0.94 | 0.54 | 0.88 | 0.88 | 0.85 |
| 50 | 0.44 | 0.59 | 0.70 | 0.58 | 0.63 | 0.79 |
| 51 | 0.09 | 0.04 | 0.17 | 0.47 | 0.48 | 0.21 |
| 52 | 0.49 | 0.72 | 0.68 | 0.83 | 0.81 | 0.36 |
| 53 | 0.52 | 0.65 | 0.69 | 0.39 | 0.56 | 0.03 |
| 54 | 0.84 | 0.91 | 0.83 | 0.85 | 0.89 | 0.85 |
| 55 | 0.37 | 0.85 | 0.81 | 0.82 | 0.85 | 0.73 |
| 56 | 0.78 | 0.86 | 0.79 | 0.81 | 0.83 | 0.68 |
| 57 | 0.78 | 0.88 | 0.77 | 0.80 | 0.82 | 0.76 |
| 58 | 0.84 | 0.91 | 0.77 | 0.83 | 0.86 | 0.87 |
| 59 | 0.47 | 0.84 | 0.71 | 0.80 | 0.81 | 0.73 |
| 60 | 0.77 | 0.81 | 0.63 | 0.90 | 0.88 | 0.65 |
| 61 | 0.47 | 0.89 | 0.85 | 0.88 | 0.89 | 0.88 |
| 62 | 0.28 | 0.67 | 0.74 | 0.86 | 0.83 | 0.61 |

Table S4. Genetic correlations for pairs of carotenoids.

| **Carotenoid** | **Carotenoid** | **Genetic correlation** | **Standard error** |
| --- | --- | --- | --- |
| α-carotene | Lutein | 0.07 | 0.03 |
| α-carotene | Zeaxanthin | 0.45 | 0.03 |
| α-carotene | β-cryptoxanthin | 0.76 | 0.02 |
| α-carotene | β-carotene | 0.37 | 0.03 |
| Lutein | Zeaxanthin | 0.08 | 0.03 |
| Lutein | β-cryptoxanthin | -0.09 | 0.03 |
| Lutein | β-carotene | 0.00 | 0.03 |
| β-cryptoxanthin | Zeaxanthin | 0.50 | 0.02 |
| β-cryptoxanthin | β-carotene | 0.17 | 0.03 |
| β-carotene | Zeaxanthin | 0.08 | 0.03 |
| Provitamin A | Lutein | -0.03 | 0.03 |
| Provitamin A | Zeaxanthin | 0.26 | 0.03 |
| Provitamin A | β-cryptoxanthin | 0.52 | 0.02 |
| Provitamin A | α-carotene | 0.66 | 0.03 |
| Provitamin A | β-carotene | 0.91 | 0.01 |

Genetic correlations with ±2 STDE is significant at *P*<0.05 level

Table S5. Hybrid means (BLUEs) for agronomic traits recorded over 12 years across locations.

| Hybrid | Number of years of testing | Anthesis | Silking | Plant height | Ear height | Husk cover | Plant aspect | Ear aspect | Grain yield |
| --- | --- | --- | --- | --- | --- | --- | --- | --- | --- |
|  |  | (days) | (days) | (cm) | (cm) | (1-5) | (1-5) | (1-5) | (kg/ha) |
| EPVA002 | 2 | 56 | 58 | 165 | 80 | 2.0 | 2.5 | 2.5 | 3792 |
| EPVA003 | 1 | 56 | 59 | 172 | 83 | 1.8 | 2.6 | 2.6 | 3349 |
| EPVA004 | 2 | 57 | 60 | 183 | 87 | 2.1 | 2.6 | 2.8 | 2709 |
| EPVA005 | 3 | 57 | 59 | 185 | 92 | 2.0 | 2.6 | 2.6 | 4039 |
| EPVA006 | 3 | 59 | 61 | 189 | 93 | 1.9 | 2.3 | 2.3 | 4483 |
| EPVA007 | 3 | 60 | 62 | 189 | 98 | 1.9 | 2.3 | 2.3 | 4377 |
| EPVA008 | 2 | 59 | 61 | 188 | 94 | 2.0 | 2.3 | 2.2 | 5288 |
| EPVA009 | 1 | 60 | 63 | 179 | 88 | 1.9 | 2.8 | 3.0 | 3442 |
| EPVA010 | 3 | 57 | 59 | 186 | 92 | 1.9 | 2.3 | 2.4 | 4816 |
| EPVA011 | 2 | 59 | 62 | 180 | 90 | 1.9 | 2.7 | 2.8 | 4035 |
| EPVA012 | 2 | 59 | 62 | 183 | 92 | 1.9 | 2.6 | 2.8 | 4234 |
| EPVA013 | 1 | 57 | 59 | 176 | 87 | 2.2 | 2.6 | 2.5 | 4172 |
| EPVA014 | 1 | 58 | 61 | 196 | 96 | 1.9 | 2.3 | 2.1 | 5690 |
| EPVA015 | 4 | 58 | 60 | 188 | 95 | 2.1 | 2.5 | 2.5 | 4893 |
| EPVA016 | 3 | 57 | 59 | 196 | 93 | 1.9 | 2.3 | 2.3 | 5201 |
| EPVA017 | 4 | 57 | 59 | 190 | 91 | 2.0 | 2.3 | 2.4 | 4945 |
| EPVA018 | 1 | 56 | 59 | 187 | 91 | 2.1 | 2.4 | 2.1 | 5538 |
| EPVA019 | 1 | 57 | 59 | 178 | 89 | 2.1 | 2.4 | 2.3 | 5155 |
| EPVA020 | 4 | 57 | 59 | 184 | 87 | 1.9 | 2.4 | 2.5 | 4707 |
| EPVA021 | 1 | 56 | 59 | 183 | 87 | 2.0 | 2.4 | 2.2 | 5408 |
| EPVA022 | 1 | 58 | 61 | 165 | 82 | 1.9 | 2.8 | 3.0 | 3111 |
| EPVA023 | 1 | 57 | 60 | 172 | 84 | 1.9 | 2.9 | 3.1 | 3214 |
| EPVA024 | 1 | 58 | 60 | 194 | 98 | 2.0 | 2.3 | 2.3 | 4804 |
| EPVA025 | 1 | 57 | 59 | 180 | 90 | 2.1 | 2.7 | 2.6 | 4101 |
| EPVA026 | 3 | 58 | 60 | 191 | 94 | 2.0 | 2.2 | 2.2 | 5315 |
| EPVA027 | 3 | 58 | 60 | 179 | 89 | 1.9 | 2.4 | 2.4 | 4917 |
| EPVA028 | 3 | 58 | 61 | 179 | 91 | 2.0 | 2.3 | 2.3 | 5238 |
| EPVA029 | 2 | 58 | 61 | 190 | 97 | 1.9 | 2.4 | 2.4 | 4758 |
| EPVA030 | 3 | 58 | 60 | 185 | 87 | 2.1 | 2.4 | 2.6 | 4797 |
| EPVA035 | 3 | 60 | 62 | 170 | 82 | 1.9 | 2.4 | 2.6 | 3480 |
| EPVA036 | 1 | 57 | 59 | 173 | 85 | 2.0 | 2.5 | 2.5 | 3862 |
| EPVA037 | 1 | 57 | 60 | 166 | 78 | 2.3 | 2.5 | 2.6 | 2903 |
| EPVA038 | 2 | 57 | 59 | 180 | 87 | 2.1 | 2.4 | 2.5 | 3442 |
| EPVA039 | 2 | 56 | 59 | 183 | 85 | 2.0 | 2.5 | 2.7 | 3807 |
| EPVA040 | 4 | 57 | 59 | 172 | 85 | 1.9 | 2.7 | 2.7 | 3747 |
| EPVA041 | 1 | 58 | 60 | 176 | 83 | 2.0 | 2.3 | 2.6 | 3490 |
| EPVA042 | 1 | 58 | 60 | 163 | 82 | 2.5 | 2.6 | 2.7 | 3157 |
| EPVA043 | 1 | 56 | 58 | 167 | 82 | 2.2 | 2.5 | 2.5 | 3373 |
| EPVA044 | 1 | 57 | 59 | 175 | 81 | 2.1 | 2.4 | 2.7 | 3590 |
| EPVA045 | 1 | 56 | 58 | 163 | 73 | 1.8 | 2.5 | 2.6 | 3620 |
| EPVA046 | 1 | 57 | 60 | 174 | 85 | 2.3 | 2.9 | 2.9 | 3703 |
| EPVA047 | 1 | 59 | 61 | 191 | 95 | 1.8 | 2.4 | 2.5 | 4032 |
| EPVA048 | 1 | 60 | 62 | 193 | 94 | 1.8 | 2.3 | 2.3 | 4620 |
| EPVA049 | 1 | 59 | 61 | 172 | 88 | 2.3 | 2.7 | 2.9 | 2632 |
| EPVA050 | 2 | 57 | 60 | 166 | 80 | 2.0 | 2.5 | 2.5 | 4013 |
| EPVA051 | 5 | 61 | 63 | 185 | 93 | 2.0 | 2.5 | 2.8 | 3719 |
| EPVA052 | 4 | 59 | 61 | 182 | 94 | 1.9 | 2.4 | 2.3 | 4554 |
| EPVA053 | 9 | 58 | 61 | 184 | 91 | 2.0 | 2.4 | 2.3 | 4617 |
| EPVA054 | 4 | 59 | 62 | 192 | 98 | 1.9 | 2.3 | 2.4 | 4426 |
| EPVA055 | 6 | 59 | 62 | 188 | 93 | 1.9 | 2.4 | 2.4 | 4352 |
| EPVA056 | 10 | 58 | 61 | 191 | 95 | 1.9 | 2.3 | 2.3 | 4964 |
| EPVA057 | 2 | 59 | 61 | 176 | 86 | 2.1 | 2.6 | 2.7 | 3852 |
| EPVA058 | 1 | 57 | 59 | 183 | 91 | 1.8 | 2.9 | 2.9 | 2667 |
| EPVA059 | 6 | 59 | 62 | 187 | 94 | 1.9 | 2.6 | 2.6 | 3968 |
| EPVA060 | 3 | 59 | 62 | 181 | 91 | 1.9 | 2.6 | 2.6 | 3445 |
| EPVA061 | 2 | 59 | 62 | 185 | 94 | 2.6 | 2.8 | 2.9 | 3658 |
| EPVA062 | 7 | 58 | 61 | 187 | 92 | 1.9 | 2.4 | 2.4 | 4570 |
| EPVA063 | 7 | 59 | 62 | 184 | 90 | 2.0 | 2.5 | 2.6 | 3958 |
| EPVA064 | 3 | 59 | 62 | 179 | 88 | 1.9 | 2.6 | 2.6 | 3703 |
| EPVA065 | 1 | 58 | 61 | 179 | 83 | 1.9 | 2.4 | 2.7 | 3915 |
| EPVA066 | 2 | 59 | 61 | 192 | 94 | 2.0 | 2.5 | 2.6 | 3712 |
| EPVA067 | 2 | 59 | 61 | 180 | 92 | 2.0 | 2.5 | 2.6 | 4022 |
| EPVA068 | 6 | 59 | 61 | 186 | 92 | 2.0 | 2.4 | 2.5 | 4482 |
| EPVA069 | 2 | 59 | 61 | 193 | 99 | 1.9 | 2.4 | 2.3 | 4549 |
| EPVA070 | 1 | 61 | 64 | 180 | 88 | 2.0 | 2.7 | 3.0 | 3024 |
| EPVA071 | 6 | 59 | 62 | 185 | 92 | 1.9 | 2.5 | 2.5 | 4218 |
| EPVA072 | 9 | 58 | 60 | 186 | 93 | 1.9 | 2.3 | 2.2 | 4936 |
| EPVA073 | 4 | 58 | 61 | 191 | 92 | 1.8 | 2.3 | 2.2 | 5022 |
| EPVA074 | 1 | 60 | 63 | 190 | 92 | 1.7 | 2.4 | 2.5 | 4239 |
| EPVA075 | 6 | 58 | 61 | 190 | 95 | 1.9 | 2.4 | 2.2 | 4945 |
| EPVA076 | 1 | 61 | 63 | 181 | 91 | 1.9 | 2.9 | 3.1 | 3112 |
| EPVA077 | 4 | 60 | 63 | 182 | 92 | 2.0 | 2.7 | 2.8 | 3739 |
| EPVA078 | 2 | 59 | 62 | 176 | 87 | 1.9 | 2.6 | 2.7 | 3683 |
| EPVA079 | 6 | 58 | 61 | 191 | 94 | 2.0 | 2.3 | 2.1 | 5158 |
| EPVA080 | 6 | 59 | 61 | 191 | 92 | 1.9 | 2.3 | 2.2 | 5045 |
| EPVA081 | 7 | 59 | 61 | 191 | 94 | 1.9 | 2.3 | 2.2 | 5144 |
| EPVA082 | 6 | 58 | 61 | 196 | 97 | 1.8 | 2.3 | 2.2 | 5155 |
| EPVA083 | 6 | 58 | 61 | 195 | 96 | 1.8 | 2.1 | 2.0 | 5505 |
| EPVA084 | 1 | 59 | 62 | 181 | 88 | 1.9 | 2.7 | 2.8 | 3445 |
| EPVA085 | 1 | 57 | 59 | 188 | 91 | 2.3 | 2.4 | 2.4 | 4409 |
| EPVA086 | 3 | 59 | 61 | 183 | 91 | 2.1 | 2.5 | 2.4 | 4064 |
| EPVA087 | 1 | 56 | 59 | 195 | 94 | 2.0 | 2.4 | 2.5 | 4645 |
| EPVA088 | 2 | 59 | 61 | 193 | 99 | 2.0 | 2.5 | 2.4 | 3754 |
| EPVA089 | 2 | 57 | 60 | 184 | 90 | 2.2 | 2.6 | 2.4 | 4182 |
| EPVA090 | 2 | 58 | 60 | 186 | 91 | 2.2 | 2.7 | 2.6 | 3834 |
| EPVA091 | 1 | 56 | 59 | 182 | 86 | 2.1 | 2.5 | 2.6 | 4403 |
| EPVA092 | 1 | 56 | 59 | 179 | 83 | 2.1 | 2.5 | 2.5 | 4367 |
| EPVA093 | 1 | 57 | 59 | 180 | 86 | 2.1 | 2.6 | 2.5 | 4480 |
| EPVA094 | 1 | 58 | 61 | 187 | 90 | 1.9 | 2.6 | 2.7 | 3050 |
| EPVA095 | 1 | 58 | 60 | 187 | 93 | 2.3 | 2.7 | 2.8 | 3802 |
| EPVA096 | 1 | 57 | 60 | 185 | 84 | 2.0 | 2.4 | 2.8 | 4379 |
| EPVA097 | 2 | 57 | 60 | 181 | 92 | 2.0 | 2.4 | 2.6 | 4662 |
| EPVA098 | 1 | 58 | 61 | 183 | 89 | 2.1 | 2.7 | 2.5 | 4430 |
| EPVA099 | 2 | 58 | 60 | 186 | 93 | 2.0 | 2.5 | 2.6 | 4462 |
| EPVA100 | 1 | 57 | 59 | 185 | 87 | 2.1 | 2.5 | 2.7 | 4473 |
| EPVA101 | 2 | 58 | 60 | 183 | 86 | 2.1 | 2.5 | 2.7 | 4400 |
| EPVA102 | 1 | 58 | 60 | 183 | 92 | 2.1 | 2.4 | 2.8 | 4331 |
| EPVA103 | 2 | 58 | 60 | 182 | 84 | 2.0 | 2.4 | 2.5 | 4595 |
| EPVA104 | 2 | 58 | 60 | 185 | 91 | 2.2 | 2.5 | 2.7 | 4394 |
| EPVA105 | 2 | 57 | 60 | 184 | 90 | 2.2 | 2.7 | 2.8 | 4406 |
| EPVA106 | 2 | 57 | 59 | 177 | 81 | 2.0 | 2.5 | 2.7 | 4464 |
| EPVA107 | 1 | 58 | 60 | 185 | 93 | 2.1 | 2.4 | 2.8 | 4264 |
| EPVA108 | 2 | 58 | 61 | 183 | 88 | 2.0 | 2.5 | 2.6 | 4660 |
| EPVA109 | 2 | 57 | 60 | 183 | 90 | 2.1 | 2.4 | 2.6 | 4666 |
| EPVA110 | 1 | 57 | 60 | 185 | 91 | 2.1 | 2.4 | 2.8 | 4390 |
| EPVA111 | 2 | 59 | 61 | 191 | 105 | 2.0 | 2.2 | 2.3 | 5044 |
| EPVA112 | 1 | 58 | 60 | 187 | 95 | 2.0 | 2.4 | 2.3 | 5333 |
| EPVA113 | 1 | 58 | 61 | 185 | 91 | 2.0 | 2.5 | 2.5 | 4790 |
| EPVA114 | 2 | 58 | 61 | 192 | 95 | 1.9 | 2.3 | 2.1 | 5335 |
| EPVA115 | 1 | 59 | 61 | 195 | 99 | 1.8 | 2.0 | 2.1 | 5090 |
| EPVA116 | 1 | 56 | 59 | 194 | 91 | 2.1 | 2.3 | 2.5 | 5176 |
| EPVA117 | 1 | 57 | 59 | 186 | 88 | 2.1 | 2.5 | 2.6 | 4713 |
| EPVA118 | 1 | 58 | 60 | 180 | 83 | 2.2 | 2.6 | 3.0 | 4417 |
| EPVA119 | 1 | 58 | 61 | 192 | 101 | 1.9 | 2.3 | 2.5 | 4848 |
| EPVA120 | 1 | 58 | 60 | 191 | 94 | 2.0 | 2.2 | 2.0 | 5152 |
| EPVA121 | 1 | 57 | 59 | 182 | 78 | 1.9 | 2.4 | 2.5 | 4593 |
| EPVA122 | 1 | 57 | 60 | 190 | 91 | 2.3 | 2.4 | 2.7 | 4712 |
| EPVA123 | 1 | 58 | 61 | 198 | 97 | 1.9 | 2.4 | 2.6 | 4425 |
| EPVA124 | 1 | 58 | 60 | 185 | 86 | 2.1 | 2.6 | 2.7 | 4681 |
| EPVA125 | 1 | 57 | 60 | 176 | 84 | 1.9 | 2.5 | 2.5 | 4838 |
| EPVA126 | 1 | 58 | 60 | 195 | 97 | 1.9 | 2.5 | 2.6 | 4943 |
| EPVA127 | 1 | 58 | 60 | 191 | 86 | 1.9 | 2.5 | 2.6 | 4740 |
| EPVA128 | 3 | 58 | 60 | 178 | 89 | 2.0 | 2.4 | 2.3 | 4979 |
| EPVA132 | 2 | 59 | 62 | 171 | 83 | 2.0 | 2.4 | 2.5 | 4059 |
| COMYH001 | 1 | 57 | 59 | 185 | 93 | 1.9 | 2.2 | 2.5 | 5277 |
| COMYH129 | 1 | 57 | 60 | 179 | 89 | 2.0 | 2.5 | 2.6 | 4225 |
| COMYH130 | 9 | 58 | 61 | 175 | 86 | 2.1 | 2.8 | 2.7 | 3861 |
| COMYH031 | 1 | 57 | 59 | 174 | 85 | 2.1 | 2.3 | 2.3 | 4993 |
| RPVA032 | 10 | 57 | 60 | 180 | 89 | 1.9 | 2.3 | 2.3 | 4754 |
| RPVA033 | 10 | 59 | 61 | 194 | 96 | 1.8 | 2.4 | 2.4 | 4148 |
| RPVA131 | 2 | 60 | 62 | 182 | 91 | 2.0 | 2.6 | 2.6 | 3845 |
| FPYC034 | 9 | 58 | 61 | 185 | 91 | 2.1 | 2.8 | 3.0 | 3681 |

Table S6. Genetic correlations for pairs of agronomic traits.

| **Trait** | **Trait** | **Genetic correlation** | **Standard error** |
| --- | --- | --- | --- |
| Anthesis | Plant height | -0.12 | 0.01 |
| Anthesis | Ear height | -0.05 | 0.01 |
| Silking | Plant height | -0.13 | 0.01 |
| Silking | Ear height | -0.07 | 0.01 |
| Ear height | Plant aspect | -0.43 | 0.02 |
| Ear height | Ear aspect | -0.30 | 0.01 |
| Ear height | Husk cover | -0.17 | 0.03 |
| Plant height | Plant aspect | -0.47 | 0.02 |
| Plant height | Ear aspect | -0.33 | 0.01 |
| Plant height | Husk cover | -0.19 | 0.03 |
| Silking | Husk cover | 0.00 | 0.02 |
| Silking | Plant aspect | 0.20 | 0.01 |
| Silking | Ear aspect | 0.27 | 0.02 |
| Anthesis | Husk cover | 0.01 | 0.02 |
| Anthesis | Ear aspect | 0.20 | 0.01 |
| Anthesis | Plant aspect | 0.27 | 0.02 |
| Husk cover | Plant aspect | 0.32 | 0.03 |
| Husk cover | Ear aspect | 0.46 | 0.04 |
| Plant aspect | Ear aspect | 0.73 | 0.02 |
| Anthesis | Silking | 0.89 | 0.00 |
| Plant height | Ear height | 0.87 | 0.01 |
| Grain yield | Anthesis | -0.22 | 0.01 |
| Grain yield | Silking | -0.23 | 0.01 |
| Grain yield | Plant height | 0.37 | 0.01 |
| Grain yield | Ear height | 0.35 | 0.01 |
| Grain yield | Husk cover | -0.19 | 0.02 |
| Grain yield | Plant aspect | -0.64 | 0.02 |
| Grain yield | Ear aspect | -0.60 | 0.01 |

Genetic correlations with ±2 STDE is significant at *P*<0.05 level
